# First impressions of Telesurgery robotic-assisted radical prostatectomy using the Edge medical robotic platform

**DOI:** 10.1590/S1677-5538.IBJU.2024.0458

**Published:** 2024-08-25

**Authors:** Marcio Covas Moschovas, Travis Rogers, Wanhai Xu, Roshane Perera, Xu Zhang, Vipul Patel

**Affiliations:** 1 AdventHealth Global Robotics Institute Florida USA AdventHealth Global Robotics Institute, Florida, USA; 2 University of Central Florida Florida USA University of Central Florida - UCF, Florida, USA; 3 Chinese PLA General Hospital Beijing China Chinese PLA General Hospital, Beijing, China

**Keywords:** Minimally Invasive Surgical Procedures, Robotic Surgical Procedures, Inventions

## Abstract

**Purpose:**

We reported, as a referral center in prostate cancer, our perspectives and experience performing Telesurgery using robotic surgery and 5G network.

**Material and methods:**

We described and illustrated the Telesurgery applications and outcomes to treat a patient with prostate cancer located 1300 kilometers away from the surgeon (Beijing-Harbin) in China. We used the Edge Medical Robot (MP1000) in November 2023 in a 71-year-old patient with Gleason 6 (ISUP 1) in 8 cores from 13, PSA of 14 ng/dL, and clinical stage cT2a. MRI described a PIRADS 5 nodule on the left peripheral zone at the base, and 20gr prostate. We described details about the connection between centers, perioperative outcomes, and our perspectives as a referral center in prostate cancer.

**Results:**

We had no delays, or problems with network connection between the centers. The procedure was performed in 60 minutes, with no intra- or postoperative complications. Estimated blood loss was 100 mL. The patient was ambulating soon after anesthesia recovery. Final pathology described a Gleason 6 (ISUP 1) involving the left base and left seminal vesicle, negative surgical margins, and no lymph node involvement (pT3bN0). The patient was continent soon after catheter removal (7 days).

**Conclusion:**

As technological progress introduced novel robotic platforms and high-speed networks, the concept of Telesurgery became a tangible reality while 5G technology solved latency and transmission concerns. However, with these advancements, ethical considerations and regulatory frameworks should underline the importance of transparency and patient safety with responsible innovation in the field.

## INTRODUCTION

In the relentless pursuit of medical and technological progress, the field of surgery has undergone a profound transition, transcending the confines of traditional operating rooms and approximating surgeons and patients from different cities and continents (1). In this scenario, Telesurgery appears as an innovative association between medicine and technology that has rewritten the history of surgical practice (2). From its landmark start with the first transatlantic procedure in 2001, where a surgeon in New York operated on a patient in Strasbourg ("Lindberg operation"), Telesurgery has become a symbol of the remarkable synergy between human expertise and digital precision (1, 3, 4). However, it was only in the last few years that a fusion of robotic surgery and a high-quality internet network enabled the expansion of Telesurgery (5–7).

Since the "Lindberg operation" that paved the way for transcontinental surgical interventions to the latest communication advancements with the 5G network, geographical limitations have been modified while we navigate through the historical landscape that redefines the current status of Telesurgery (8). In the past three years, several groups have described different long-distance procedures in animal models and humans with optimal rates of success (9, 10).

Telesurgery brings tremendous humanitarian potential to underserved areas with restricted access to surgical specialties (11). With this technology, patients can be treated by an expert located thousands of kilometers away, and the same surgeon can operate on patients from different cities or countries on the same day. In this scenario, after having experience with several robotic platforms (12,13), we described our perspectives and experience performing a robotic-assisted radical prostatectomy using Telesurgery with the Edge robot.

## MATERIAL AND METHODS

We performed a Telesurgery robotic-assisted radical prostatectomy on a 71-year-old patient with Gleason 6 (ISUP 1) in 8 cores from 13, PSA of 14 ng/dL, and clinical stage cT2a. MRI described a PIRADS 5 nodule on the left peripheral zone at the base, and 20gr prostate. We described details about the connection between centers, perioperative outcomes, and our perspectives as a referral center in prostate cancer.

### Network technology and data management

In Telesurgery, network technology is essential to guarantee the feasibility and success of the surgical procedure. The data transmission is performed with 4G or 5G internet technology with or without exclusive optical fiber (wired transmission) support as a backup in case of issues with the Wi-Fi connection (14, 15). In this scenario, several variables are monitored during the procedure while the remote surgeon is operating. Every 2 minutes, the roundtrip network latency is calculated to ensure the transmission quality because, usually, delays higher than 100 to 300ms could compromise the synchrony between surgeon and remote patient (16). Round trip latency refers to the time it takes for data to travel from the remote operating console to the surgical site and back, encompassing the entire communication cycle. In Telesurgery, where precision and real-time response are imperative, understanding and minimizing roundtrip latency becomes vital.

Time-to-live (TTL) represents the duration or maximum number of hops a data packet can undertake before being discarded. This parameter plays a pivotal role in maintaining the integrity and efficiency of communication between the surgical site and the remote operating console. In this context, understanding the significance of TTL becomes essential for protecting the information flow and ultimately ensuring the success and precision of remote surgical interventions.

Frame loss (expressed in dB/km or dB/m) is another crucial parameter to establish the performance of an optical fiber. In a machine vision system, the main consideration is to guarantee the stable and swift transmission of each frame's image to the computer equipment. However, due to frequent issues arising from inadequate hardware and software compatibility, image data loss, commonly called dropped frames (Frame loss), occurs during transmission. This frame loss manifests as abnormal data processing, display results freezing, and image faults.

In our experience, the connection between Beijing (Chinese PLA General Hospital) and Harbin (Harbin Medical University Cancer Hospital) used a 5G network and OTN (optical transport network) dedicated line with low latency, large bandwidth, high reliability, and high security.

### Robotic platform and surgical technique

**Figure 1:**
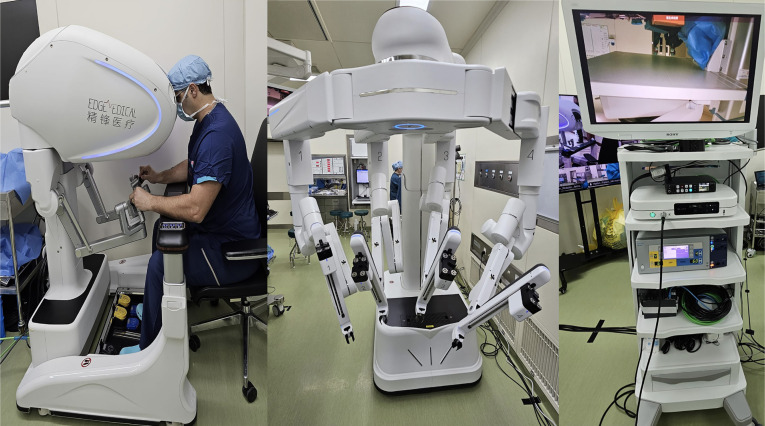
Edge robot console and multiport patient cart.

In addition to the transmission technology, a robotic platform able to connect and perform Telesurgery is also needed. We used the Edge Medical robotic platform MP1000 (Shenzhen Edge Medical Co., Ltd., Shenzhen, China), a multiport platform composed of four arms attached to a single tower (Figure-1) (17).

**Figure 2:**
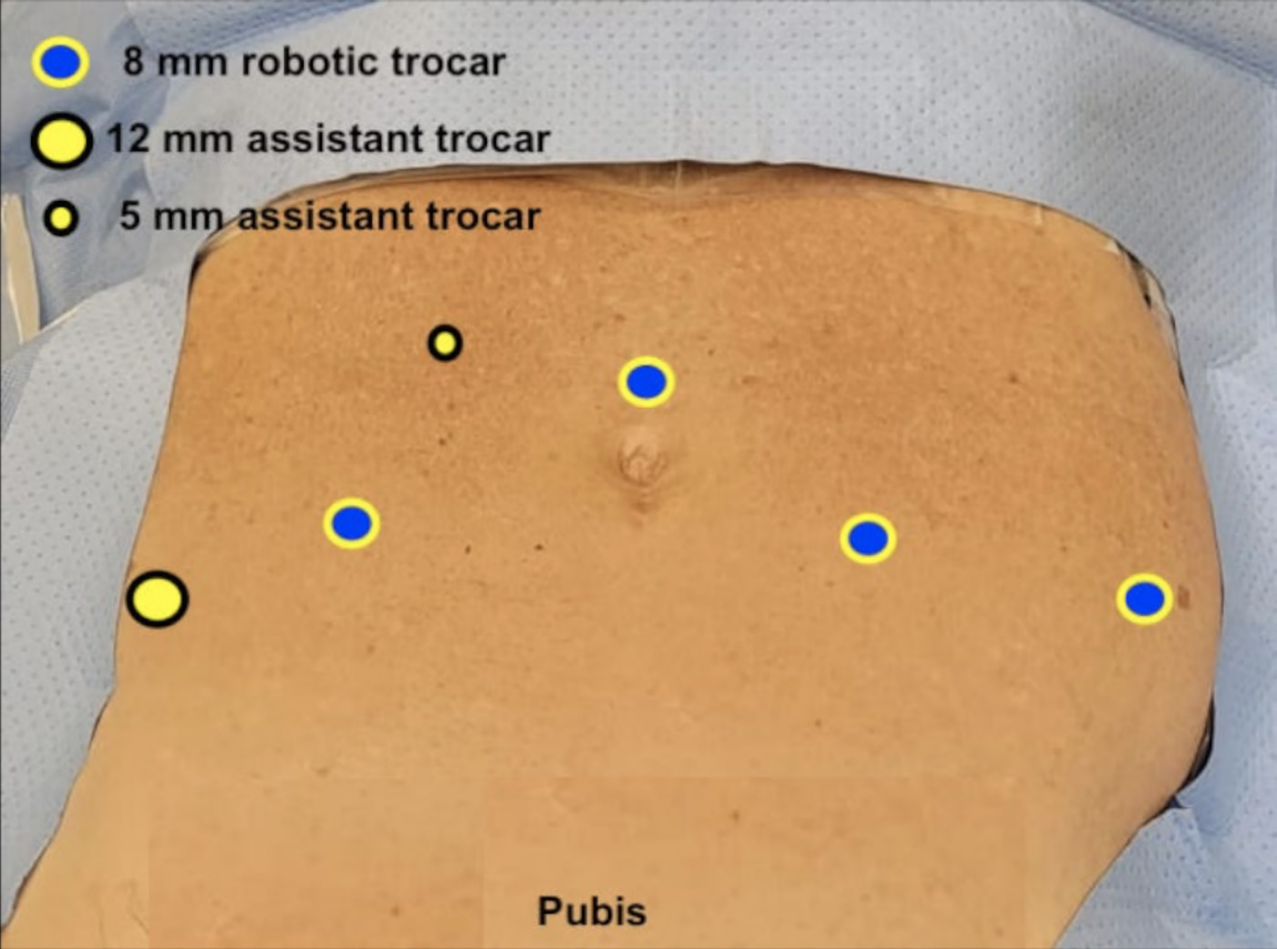
Trocar placement.

**Figure 3:**
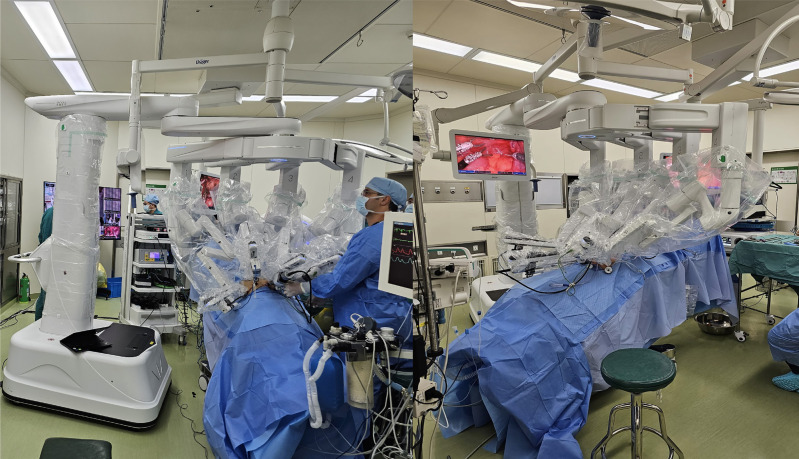
Operation Room setup during surgery.

The trocar placement followed a conventional multiport position with four robotic trocars and two additional trocars for the assistant (Figure-2). This platform provides three instruments with 8mm and a 3D endoscope with 8mm. After placing the trocars, the robot is docked (Figure-3), the instruments are placed (Scissors, Prograsp, and Maryland), and the procedure follows our conventional robotic-assisted radical prostatectomy technique (18–23) with the following sequence:

1. Bladder detachment
2. Anterior Bladder neck dissection
3. Posterior Bladder neck dissection
4. Seminal vesicles control with athermal technique and Hem-o-lok clips
5. Posterior prostate dissection and nerve-sparing between Denonvilliers layers
6. Lateral prostate dissection communicating lateral and posterior planes of the prostate
7. Prostate arterial pedicle control with Hem-o-lok clips
8. Minimal Apical Dissection and DVC control with running suture
9. Urethra division and hemostasis
10. Posterior reconstruction (Rocco's stitch) and Anastomosis with barbed suture
11. Pelvic Lymph node dissection

### Telesurgery Logistics between centers

We performed a robotic-assisted radical prostatectomy between Beijing (Chinese PLA General Hospital) and Harbin (Harbin Medical University Cancer Hospital). All telesurgery procedures are approved by the Institutional review board and administrative bodies of both centers involved in the patient care. The patient was located in Harbin, while the main surgeon was in Beijing, approximately 1300 kilometers away.

**Figure 4:**
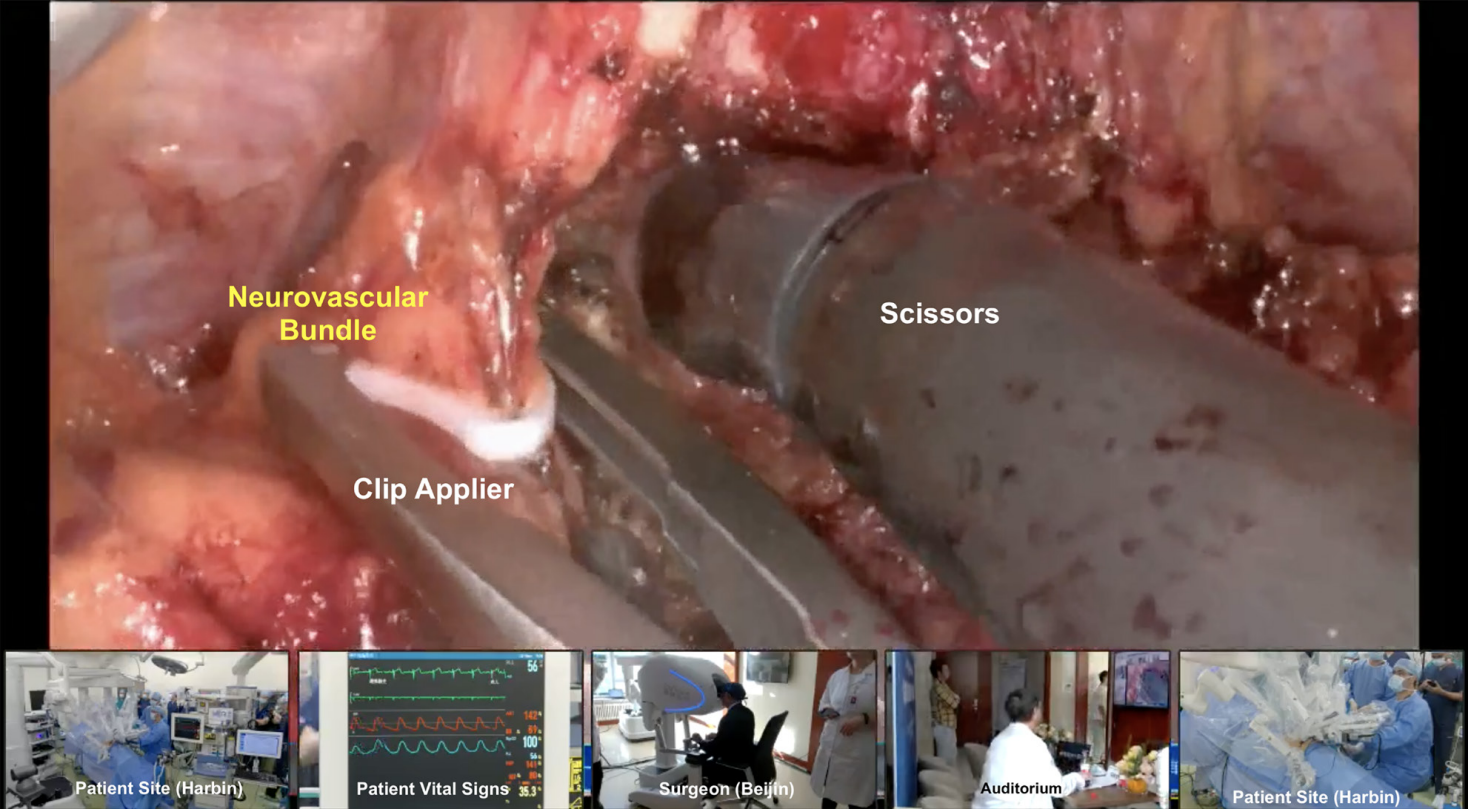
Transmission setup showing surgeon, remote team with the patient, patient vital signs, and auditorium.

It is important to note that, on the patient side of the transmission, the tableside assistant was also an expert robotic surgeon (MCM) who would finish the procedure in case of any transmission issues. This is crucial to guarantee patient security and optimal outcomes in case of technological problems during the surgery, especially during the implementation of Telesurgery. The imaging and audio communication between both surgeons is smoothly performed like a conventional robotic surgery, with a microphone and speakers on the surgeon's console and assistant's room. We had no pertinent delays in the data transmission; audio and video were not compromised at any moment of the surgery. During the broadcast, we had cameras filming the surgeon, patient, vital signs monitor, and staff from both centers (Figure-4).

## RESULTS

### Patient demography and perioperative data

The digital rectal exam described a T2a on the left side. The patient had a preoperative MRI showing a PIRADS 5 lesion on the left apex and mid (Peripheral zone). The procedure was performed in 60 minutes with no intra- or postoperative complications and an estimated blood loss of 100 mL. The patient was ambulating soon after anesthesia recovery (approximately 4 hours after surgery). However, he stayed in the hospital for four days due to the postoperative routine of the local team. The Foley catheter was removed seven days after surgery, and the patient was continent soon after catheter removal. We define continence as the full capacity to hold the urine (no use of pads) after removing the catheter.

Final pathology described a Gleason 6 (ISUP 1) involving the left seminal vesicle, negative surgical margins, and no lymph node involvement (pT3bN0).

### Data transmission and network details

**Figure 5:**
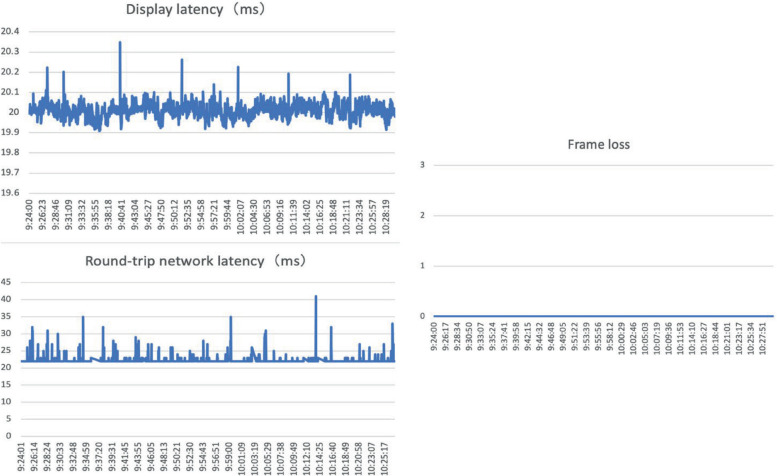
Connection details showing the Display Latency, Frame loss, and Round-trip network latency.

The network was collected as the median value and interquartile range (IQR) of transmission data. The Roundtrip network latency was 22 (22–22) milliseconds, Time to Live (TTL) was 64 (64–64) bits, and no Frame Loss in decibels per kilometer (dB/km) was recorded. Figure-5 illustrates a graphical analysis of these variables.

## DISCUSSION

The current scenario of Telesurgery integrates medical expertise and advanced robotic and telecommunication technology, with many advantages that have reshaped the landscape of surgical practice (2). One of its primary merits lies in the democratization of specialized surgical care, as it allows skilled surgeons to perform procedures remotely, overcoming geographical barriers and extending their reach to underserved or remote regions. This not only enhances accessibility but also facilitates timely interventions, particularly in emergencies. Additionally, Telesurgery contributes to the globalization of medical expertise, enabling collaboration between renowned surgeons in challenging cases, regardless of their physical locations (24). The precision and dexterity of robotic systems utilized in Telesurgery enhance the surgeon's capabilities, improving patient outcomes and minimizing the invasiveness of procedures. Moreover, the technology facilitates real-time consultation and guidance, promoting continuous learning and skill development within the medical community. As Telesurgery continues to evolve, its advantages promise to transform the traditional paradigms of surgery, making specialized care more accessible, efficient, and globally interconnected.

We described our initial Telesurgery experience in patients undergoing robotic-assisted radical prostatectomy. After years of using robotic technology to operate on patients in the Urology field, our first impression of remote surgery was very optimistic. Initially, our major concern was the potential surgery transmission and communication issues between both centers 1300 km apart. Therefore, to assist with the procedure, we sent an experienced robotic surgeon from our team to the patient site who was able to finish the surgery locally in case of any technical problems. However, in our experience, we could not detect transmission delays or any technological issues that could compromise the patient's care and the optimal quality of the surgery. At all times, even in specific moments that need synchrony between surgeon and assistant, such as prostate pedicle clipping, we could perform our surgical technique and communicate in the same way we do in our robotic surgery routine without imagining our audio issues.

This synchrony of audio and video between the console and the robotic platform is only possible with optimal connection provided by the 5G, optic fiber, or both combined (2, 25). Telesurgery transmission is a pivotal component in remote surgical interventions, where fast communication between the surgical site and the remote operating console is essential. The transmission process involves real-time data exchange over a network, including high-definition images and vital surgical information. The reliability and efficiency of this data transfer are critical for ensuring the precision and success of telesurgery procedures. Time to Live (TTL) plays a crucial role in selecting the duration or maximum number of hops a data packet can undergo before potential loss. Round trip latency, encompassing the time taken for data to travel from the remote console to the surgical site and back, directly influences the responsiveness and real-time nature of the surgical interaction. The continuous advancement of technology in telesurgery transmission not only addresses these challenges but also holds the promise of further optimizing the remote surgical experience, pushing the boundaries of what is achievable in remote surgical routine.

During the Telesurgery implementation, learning phase, and maintenance, we believe designing a surgical program focused on patient safety and ethical standards is crucial. As we navigate this new technological approach, we should avoid potential negative impacts on the patient's safety and operative outcomes. Therefore, we believe the first step is to provide a local team that is proficient in robotic surgery and can place the trocars, dock the robot, insert the instruments, and even finish the surgery in case of connection issues with the main surgeon. In addition, in some cases of patients with previous surgeries and bowel adhesions, the local team should have the expertise to perform the lysis of adhesions before placing the trocars. Therefore, in the current Telesurgery stage, we still need considerable training and expertise from the local team side of transmissions to provide optimal patient care.

It is crucial to acknowledge that, before performing the case, extensive preoperative testing ensured optimal connectivity. Successful telesurgery involves a collaborative community of experts to optimize connectivity and uphold ethical standards for the best patient outcomes. Telesurgery demands significant collaboration from government bodies and a diverse community of specialists. No single entity can succeed independently; it requires collective effort with coordination among robotic companies, surgeons, patients, patient advocates, telecom companies, hospital teams, administration, licensing committees, medical societies, governing bodies, healthcare payors, and legal experts. Without a clear understanding and coordination of these components, telesurgery risks causing harm and likely will fail over time. In this scenario, our collaborative community described the 10 commandments of a safe and ethical exploration of telesurgery (26–28).

Besides the data transmission, security, and robotic surgery expertise, it is crucial to have a robotic platform with connectivity capacity to perform Telesurgery. In our remote surgery experience, the case was performed using the MP1000 (multiport) robot from Edge Medical, which has similar port placement, docking, instrumentation, and operative performance compared to the conventional multiport platform in the market in which we have thousands of cases of experience. We could replicate and maintain all steps of our surgical technique from the trocar placement until the end of the surgery. This is crucial to maintain our surgical standards and guarantee optimal performance, patient security, and satisfactory operative outcomes.

During the case, the surgeon on the console (VP) experienced no delays in moving the instruments or communicating with the remote assistant (MCM). The sensation was identical to our routine cases where the surgeon and assistant are working in the same room. The machine's performance during different surgical steps of this remote surgery was consistent. We detected no delays or issues when swapping the 3rd and 4th arms or adjusting the scope 30 up or down. In scenarios of increased delays, it is possible to visualize a delay difference while pressing the energy pedal and watching the tissue reaction on the console screen. In our experience, the energy was applied instantaneously to the tissues upon using the bipolar or monopolar pedals, just like in non-telesurgical cases. Additionally, the needle drivers, with their wrist-like angulation, enabled us to perform the anastomosis in the same conventional manner as with other platforms. In this scenario, we believe that recent advancements in data transmission associated with the new robotic platforms in the market enabled Telesurgery to become a reality in different countries, which implicates a huge humanitarian potential to further approximate surgeons and patients while providing a step forward on the healthcare quality, especially on underserved areas.

## CONCLUSIONS

The future of Telesurgery holds the transformative potential to redefine the status of surgical practice in unprecedented ways. As technology advances, we anticipate increasingly sophisticated robotic systems with enhanced precision and sensory capabilities, offering surgeons an augmented range of motion and improved real-time feedback. Furthermore, virtual and augmented reality integration may engage surgeons in immersive environments, enhancing their situational awareness and dexterity during remote procedures. The advent of 5G technology promises to address latency issues, ensuring faster and more reliable data transmission for optimal telesurgical experience. Additionally, the global collaboration among medical experts, surgical societies, and healthcare authorities will likely intensify, promoting collaborations and expertise that transcend geographical boundaries. Ethical considerations and regulatory frameworks will continue to evolve with technological progress, emphasizing the need for transparency, patient safety, and responsible innovation.
